# Insulin modulates the paired-pulse plasticity at glutamatergic synapses of hippocampal neurons under hypoinsulinemia

**DOI:** 10.3389/fncel.2023.1132325

**Published:** 2023-03-21

**Authors:** Mariia Shypshyna, Oksana Kolesnyk, Svitlana Fedulova, Nickolai Veselovsky

**Affiliations:** Department of Neuronal Networks, Bogomoletz Institute of Physiology, Kyiv, Ukraine

**Keywords:** hypoinsulinemia, hippocampus, glutamatergic neurotransmission, postsynaptic currents, paired-pulse plasticity

## Abstract

Hypoinsulinemia is a pathological consequence of diabetes mellitus that can cause a number of complications of the central and peripheral nervous system. Dysfunction of signaling cascades of insulin receptors under insulin deficiency can contribute to the development of cognitive disorders associated with impaired synaptic plasticity properties. Earlier we have shown that hypoinsulinemia causes a shift of short-term plasticity in glutamatergic hippocampal synapses from facilitation to depression and apparently involves mechanisms of glutamate release probability reduction. Here we used the whole cell patch-clamp recording of evoked glutamatergic excitatory postsynaptic currents (eEPSCs) and the method of local extracellular electrical stimulation of a single presynaptic axon to investigate the effect of insulin (100 nM) on the paired-pulse plasticity at glutamatergic synapses of cultured hippocampal neurons under hypoinsulinemia. Our data indicate that under normoinsulinemia additional insulin enhances the paired-pulse facilitation (PPF) of eEPSCs in hippocampal neurons by stimulating the glutamate release in their synapses. Under hypoinsulinemia, insulin did not have a significant effect on the parameters of paired-pulse plasticity on neurons of PPF subgroup, which may indicate the development of insulin resistance, while the effect of insulin on PPD neurons indicates its ability to recover the form normoinsulinemia, including the increasing probability of plasticity to the control level in of glutamate release in their synapses.

## 1. Introduction

Hypoinsulinemia is one of the diabetic syndromes and causes a number of complications of the central and peripheral nervous system functioning. Under type 1 diabetes mellitus this condition can manifest itself by decreased production of insulin by pancreatic β-cells caused by their massive death, and under type 2 diabetes mellitus following overstimulation of the insulin secretory machinery of the β -cell for compensation of permanent hyperglycemia. The brain is a highly insulin-sensitive organ (Zhao et al., 2004) with high expression of insulin receptors predominantly in the hippocampus, neocortex, cerebellum, etc. (Fernandez and Torres-Alemán, 2012). Hence, many cognitive impairments associated with diabetes can also occur due to dysfunction of insulin receptor signaling cascades under hypoinsulinemia.

Recently much attention has been devoted to the role of insulin-dependent signaling in the regulation of neurogenesis and synaptic plasticity in the hippocampus and its effect on memory and learning processes. Various positive effects of insulin have been described, such as the neurotrophic action on both differentiated neurons and neuronal stem cells (Kleinridders et al., 2014), stimulation of synaptogenesis (Chiu et al., 2008) and promotion of synaptic plasticity (Huang et al., 2004; Grillo et al., 2015; Spinelli et al., 2019; Zhao et al., 2019). Insulin induces both presynaptic and postsynaptic forms of neuronal plasticity at hippocampal synapses. This hormone stimulates the proliferation and metabolism of insulin-sensitive glia (Heni et al., 2011) and therefore may affect the functional state of neurons and their synaptic connection properties. Interestingly, insulin is a modulator rather than an “inducer” of synaptic plasticity, and multiple sites of action are responsible for the effect of insulin on synaptic plasticity (Mainardi et al., 2015). Thus, dysfunction of insulin signaling pathways caused by decline of insulin levels can lead to impairment of cognitive processes under different pathological conditions.

Since hypoinsulinemia may affect the function of many insulin-sensitive organs, the primary culture of rat hippocampal neurons can be successfully used to determine the effect of insulin on the functioning of hippocampal synapses and their plasticity. Such approach allows to eliminate the numerous modulatory effects of insulin on some other organs and body systems.

The role of insulin in synaptic plasticity at hippocampus has been studied earlier (Lee et al., 2011; Mainardi et al., 2015, Zhao et al., 2019), however, the locus of short-term plasticity expression and the improving role of insulin in presynaptic release of glutamate in these neurons remain unclear, especially under conditions of previous insulin deprivation. The aim of this study was to investigate the insulin effects on modulating of paired-pulse plasticity at glutamatergic synapses of hippocampal neurons under hypoinsulinemia model.

## 2. Materials and methods

### 2.1. Ethical approval

All experimental procedures were performed in accordance with international principles of the European Convention for the protection of vertebrate animals used for experimental and other scientific purposes, Strasburg, 1986; the Law of Ukraine “On protection of animals from cruelty” and approved by the Animal Care Committee of Bogomoletz Institute of Physiology.

### 2.2. Hippocampal neurons culture preparation

Primary hippocampal neuronal culture from neonatal Wistar rats was prepared as described previously (Fedulova et al., 1999) with some modifications. Briefly after decapitation the rat hippocampus was removed, dissected into segments and incubated in 0.05% trypsin (type II) solution during 10 min (t = 23–25^°^C). Then, the hippocampal segments were washed with culturing solution containing Eagle’s modified medium (MEM) 9.6 g/l, 10% horse serum, 2.2 g/l NaHCO_3_, 103 nM insulin, penicillin 25 U/ml and streptomycin 25 μg/ml. The concentration of insulin for hippocampal neurons culturing was quite consistent with the physiological insulin levels, that can be found in brain tissues (Schechter et al., 1992; Duarte et al., 2012; Ghasemi et al., 2013). After mechanical dissociation (by Pasteur’s pipettes) in culturing solution, hippocampal neurons were plated into the previously poly-L-ornithine-coated coverslips in Petri dishes at cell density approximately 30,000 cm^–2^. Further, the neurons were incubated at 37^°^C at 5% CO_2_ in culturing solution during 3 days, and after that 5 μM cytosine-A-D-arabinofuranoside was added for 24 h to the culture medium to reduce glia proliferation.

To simulate hypoinsulinemia, the cultured (16–20 days *in vitro*) hippocampal neurons were incubated in insulin-free media for 4 days [the time of manifestation of a significant effect of insulin deprivation on synaptic transmission according to our previous studies (Shypshyna et al., 2021)]. Electrophysiological experiments were carried out using cells after 20–24 days in culture.

### 2.3. Electrophysiology

Using the patch-clamp method under “whole cell” configuration and the method of local extracellular electrical stimulation of single presynaptic axon, the glutamatergic evoked excitatory postsynaptic currents (eEPSCs) in hippocampal neurons were recorded and analyzed. To obtain the best control of intracellular potential and to reduce “dendritic filtering” of eEPSCs, we performed axonal stimulation in close proximity to the soma of the postsynaptic neuron. Local electrical stimulation was performed by rectangular voltage pulses of negative polarity with a duration of 0.4 ms and frequency of 0.5 s^–1^, that were supplied through stimulation micropipette (inner diameter of about 2 μm) filled with a standard extracellular solution and connected to the outlet of the ISO-Flex isolated output stimulator (AMPI, Israel). The amplitude of the voltage at stimulating pipette input varied from 30 to 40 V. The relationship between the output voltage (0–30 V) and the potential close to the mouth of the stimulating pipette was linear. The position of stimulating pipette was set considering the diameter zone of effective potential shift (Fedulova et al., 1999), in close (1–2 μm) proximity to the presumable axon.

To estimate the short-term plasticity a paired-pulse ratio (PPR) was calculated using the peak amplitudes of two consecutive EPSCs (interpulse interval 50 ms) by dividing the mean amplitude of the 2nd eEPSC by that of the 1st eEPSC. The period between one pair of stimuli and the next pair was 3 s, which was sufficient for full recovery of eEPSCs. The coefficients of variation values for the 1st and 2nd eEPSCs amplitudes (CV1 and CV2) were compared to estimate the average CV ratio (CV2/CV1).

All experiments were carried out at 20–22^°^C. Extracellular bath solution contained (in mM): NaCl 140; KCl 3; CaCl_2_ 2; MgCl_2_ 2; glucose 6; HEPES 20 (pH was adjusted to 7.4 with NaOH). To reduce the GABA- and glycinergic neurotransmission in culture 1 μM strychnine and 10 μM bicuculline were always added to the bath solution. The hippocampal neuron was voltage-clamped at –70 mV. Patch pipettes from borosilicate glass (WPI, USA) with tip inner diameter 1–1.5 μm were filled with intracellular solution containing (in mM): K-gluconate 155; EGTA 0.5; MgCl_2_ 1; HEPES 20 (pH was adjusted to 7.2 with KOH). Exchange of external solution and drugs application was performed using a rapid-change system (2 ml per min). All drugs were obtained from Sigma.

To control the series resistance and quality of voltage clamping at hippocampal neurons throughout the experiments the time constant of capacitive current in response to rectangular hyperpolarizing stimulus (10 ms duration, –10 mV amplitude) and amplitude of leakage current were monitored. Data were not included into analysis, if significant variation (>20%) of these parameters occurred during experiment.

The experimental setup was constructed by the staff of Bogomoletz Institute of Physiology, Kyiv, Ukraine. The recorded currents were filtered at 5 kHz, digitized at 10 kHz and stored in a personal computer for display and analysis using two EPC-8 amplifiers (“HEKA,” Germany), DigiData 1322A analog-to-digital converter interface, and the WinWCP v3.9.6 (University of Strathclyde, UK) and pClamp 9.0 (Axon Instruments) software.

### 2.4. Statistical analysis

A simple binomial model was used according to the previously described methods (Sola et al., 2004) to calculate the probability of glutamate release (*p) and* the value of quantum content (*m):*


$$p=1-\frac{M\,{\text{CV}}^{2}}{q\,\left(1+C\,V^{2}\right)};m=M/q$$

where *M* and CV^2^ are the mean and the coefficients of variation of the 1st eEPSCs in pairs under paired-pulse stimulation; *q* and cv^2^ are the mean and the coefficients of variation of miniature synaptic currents (mEPSCs). The glutamatergic mEPSCs were measured in extracellular low Ca^2+^/high Mg^2+^ solution (Isaacson and Walmsley, 1995) containing 0,5 mM Ca^2+^, 10 mM Mg^2+^ and 0,25 μM TTX (Fedulova et al., 1999).

All data are presented as mean ± SEM. Statistical analysis of experimental data were performed by the paired and unpaired Student’s *t*-test. In all cases, *n* refers to the number of hippocampal neurons. All data were tested for normality using the Shapiro–Wilk test. Two-way ANOVA test with *post-hoc* Bonferroni tests was used where needed (factors: culturing conditions: normo-/hypoinsulinemia, insulin effect: control/insulin addition).

## 3. Results

**FIGURE 1:**
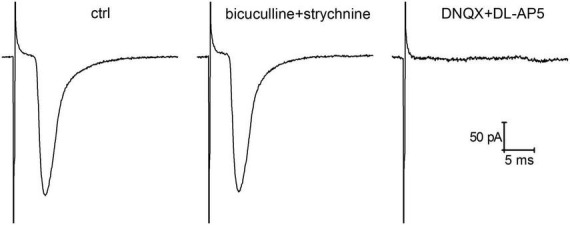
Glutamatergic evoked glutamatergic excitatory postsynaptic currents (eEPSCs) recorded in cultured hippocampal neurons following local extracellular electrical stimulation of single presynaptic axon. The represented traces of eEPSCs (averaged of 50 sweeps) demonstrate the action of GABA (gamma-aminobutyric acid)- and glycinergic neurotransmission blockers (1 μM strychnine and 10 μM bicuculline) and the effects of ionotropic glutamate receptor antagonists (10 μM DNQX and 10 μM DL-AP5) on the amplitude of eEPSCs.

In order to determine whether insulin affects presynaptic expression of glutamatergic plasticity at hippocampal synapses under hypoinsulinemia, the standard paired-pulse plasticity paradigm was applied. Using the method of single presynaptic axon stimulation combined with whole cell patch-clamp recording we analyzed the monosynaptic glutamatergic eEPSCs under paired-pulse stimulation at 50 ms inter-stimulus intervals. The analyzed monosynaptic eEPSCs were mediated by activation of ionotropic glutamate receptors and were entirely abolished by application of 10 μM DNQX with 10 μM DL-AP5 (Figure 1). Addition of GABA- and glycinergic receptor blockers (1 μM strychnine and 10 μM bicuculline) did not alter the amplitude and kinetics of eEPSCs.

**FIGURE 2:**
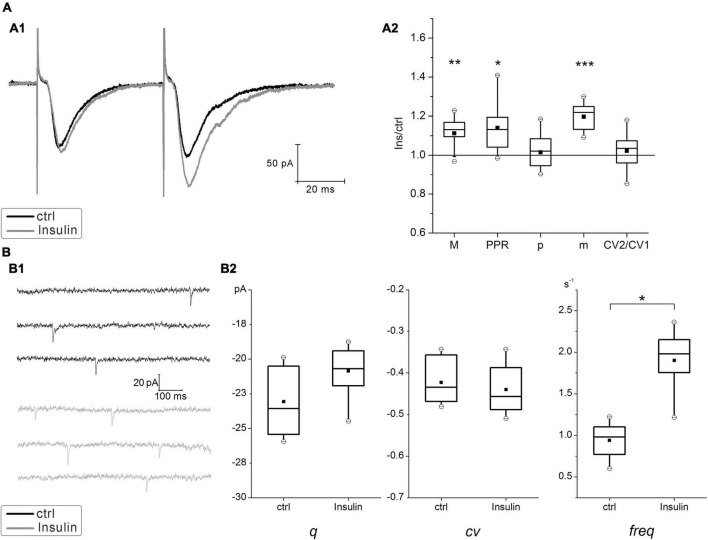
Effect of insulin on glutamatergic excitatory postsynaptic currents (EPSCs) at hippocampal neurons cultured under normoinsulinemia conditions. **(A)** Paired-pulse plasticity of evoked glutamatergic excitatory postsynaptic currents (eEPSCs) changes under insulin action. **(A1)** Sample traces of eEPSCs pairs in control (ctrl) and after 100 nM insulin application (Insulin) in the same single cell (averaged of 30 sweeps). **(A2)** The box charts show average changes of the 1st eEPSCs amplitudes (M) in the pairs, the coefficient of variation (CV), the paired-pulse ratio (PPR), the release probability (*p*) and the quantal content (*m*) after insulin addition compared with the relative parameters measured at the same synapses in control (taken as 100%). Statistical significance is indicated by: *P < 0.05; **P < 0.005; ***P < 0.001 compared with control (paired Student’s paired *t*-test, *n* = 15). **(B)** Insulin effects on miniature EPSCs (mEPSCs). **(B1)** Sample traces of mEPSCs measured at the same neurons before (ctrl) and after (Insulin) extracellular insulin application. **(B2)** Comparison box charts of mEPSCs amplitude (quantal size, *q*), the coefficient of variation of mEPSCs (cv) and mEPSCs frequency (freq) before (ctrl) and after (Insulin) insulin addition. Statistical significance is indicated by: *P < 0.05 compared with control (Student’s paired *t*-test, *n* = 7).

In hippocampal neurons cultured at normal insulin concentration (normoinsulinemia), the paired stimulation of the single presynaptic axon usually elicited the paired-pulse facilitation (PPF) of glutamatergic eEPSCs (Figure 2A). Application of insulin at concentration of 100 nM for 4 min to these neurons lead to slight raising of the mean amplitudes of eEPSCs (ratio 1.1 ± 0.02, *P* < 0.005; *n* = 15) and to the increase of PPR from 1.15 ± 0.01 to 1.25 ± 0.01 (*P* < 0.05; *n* = 15), while there were no statistically significant changes in the CV ratio of eEPSCs under the insulin action (in control CV1 = –0.36 ± 0.03 and CV2 = –0.37 ± 0.03; after insulin addition CV1 = –0.33 ± 0.03 and CV2 = –0.34 ± 0.03; *P* = 0.16; *n* = 15). It should be clarified, that during the experiment the neurons of different cultures were kept in the extracellular solution before insulin application for approximately the same time. We have identified *de novo* insulin effect on paired-pulse plasticity, therefore each neural network was not reused to avoid the cumulative effect of insulin action during repeated washouts.

To verify whether insulin modulates the release probability at hippocampal synapses we estimated the amplitude and frequency of glutamatergic mEPSCs in a distinct series of experiments. Insulin did not change the amplitude of mEPSCs (Figure 2B), which would reflect an effect of single quanta release, but slightly increased its frequency (from 0.97 ± 0.15 to 1.86 ± 0.18 s^–1^; *P* = 0.005; *n* = 7), that is associated with the enhancement of basal neurotransmitter release. These results are consistent with the previous reports about stimulating effect of insulin on the increase of mEPSCs frequency (Lee et al., 2011).

Use of simple binomial statistics to calculate the probability of glutamate release (*p*) and quantal content (*m*) values showed a significant increase of *m* approx. by 1.2 times (*P* < 0.005; *n* = 15), while *p* did not change significantly in insulin (0.64 ± 0.01 and 0.68 ± 0.01) (Figure 2A). This suggests that enhancing effect on glutamatergic eEPSCs might be realized through the mechanisms different from potentiation of presynaptic release.

**FIGURE 3:**
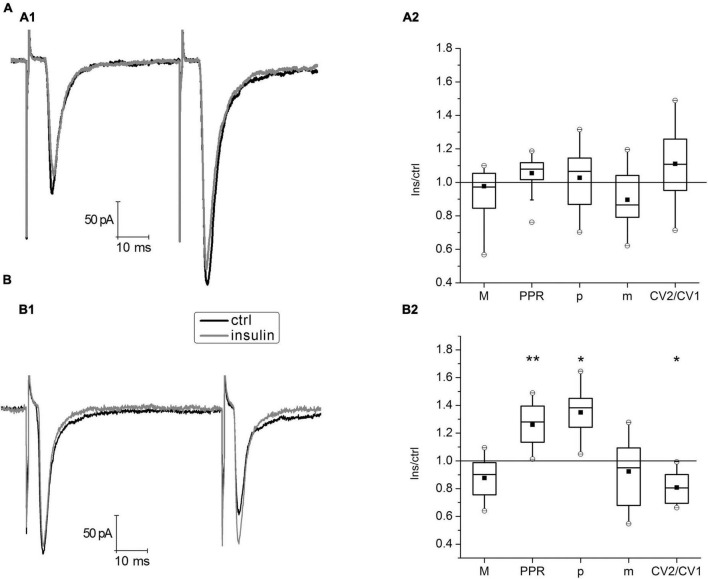
Insulin action on paired-pulse plasticity of evoked glutamatergic excitatory postsynaptic currents (eEPSCs) at glutamatergic synapses of hippocampal neurons under hypoinsulinemia. **(A)** Paired-pulse facilitation (PPF) under 100 nM insulin addition. **(A1)** Average traces of eEPSCs pairs in control (ctrl) and after insulin application (Insulin) in the same single cell. **(A2)** The box charts show average changes of PPF parameters amplitudes (M), coefficient of variation (CV), paired pulse ratio (PPR), release probability (*p*), and quantal content (*m*) (same as in Figure 1) under insulin action compared with those measured before insulin addition (control) at the same synapses (taken as 100%). Differences of corresponding values with those in control were not significant (Student’s paired *t*-test, *P* > 0.17, *n* = 17). **(B)** Paired-pulse depression (PPD) after 100 nM insulin addition. Sample traces of eEPSCs pairs **(B1)** and summary plot of PPD parameters changes **(B2)** are presented similarly, to those in **(A)**. Statistical significance is indicated by: *P < 0.05; **P < 0.005 compared with control (Student’s paired *t*-test, *n* = 16).

To simulate hypoinsulinemia conditions, mature hippocampal cell cultures 16–20 DIV were placed into an insulin-free medium for 4 days, after that electrophysiological experiments were carried out. The experimental conditions corresponded to those described for cultures of the normoinsulinemia group. In hypoinsulinemia, glutamatergic hippocampal synapses were divided into two subgroups depending on the expression of the paired-pulse plasticity form (Figures 3A, B). In the PPF subgroup (*n* = 17) insulin application for 4–6 min did not cause significant changes in the parameters of PPR, CV2/CV1 ratio and binomial parameters *p* and *m*. In the PPD subgroup, insulin significantly increased the value of PPR from 0.77 ± 0.005 to 0.97 ± 0.006 (*P* < 0.005; *n* = 16) and decreased the CV2/CV1 ratio from 1.67 ± 0.02 to 1.3 ± 0, 01 (*P* < 0,05; *n* = 16), that may indicate an increase of release probability at glutamatergic synapses on these neurons. Additional analysis of binomial parameters confirmed these results: *p* increased by 1.3 times, while *m* did not change. In both subgroups, the insulin action on hippocampal neurons cultured under hypoinsulinemia conditions did not cause significant changes of their eEPSCs amplitudes (1st eEPSC in a pairs). We compared the 1st eEPSC in the PPF and PPD subgroups: mean values did not differ [M (PPF) = –131.5 ± 32.2 pA; M (PPD) = –100.1 ± 48.0 pA; *p* = 0.6, unpaired Student’s *t*-test]. In addition, the PPR values in the PPD subgroup of hypoinsulinemia after insulin addition did not differ significantly from those measured in the control of normoinsulinemia (*p* > 0.05; two-way ANOVA), while control PPR values in normoinsulinemia and in the PPD subgroup of hypoinsulinemia significantly differed (*p* < 0.05; two-way ANOVA).

The present results are consistent with the previous data regarding presynaptic modulation of plasticity in glutamatergic synapses of hippocampal neurons under insulin action (Ferrario and Reagan, 2018), when the blockade of insulin receptors leads to a decrease in the efficiency of excitatory neurotransmission due to a decrease of presynaptic release probability of glutamate.

## 4. Discussion

Insulin signaling in the hippocampus has been targeted to improve impaired cognitive activity associated with diabetes mellitus and obesity (Fernandez and Torres-Alemán, 2012). Insights into the underlying electrophysiological mechanisms of insulin-mediated changes in synaptic transmission and plasticity may further refine treatment efficacy. Our studies demonstrate that insulin application to hippocampal neurons cultured at normoinsulinemic conditions slightly increases the amplitude of the glutamatergic eEPSCs and, accordingly, their quantum content (*m*), while no significant changes of the release probability (*p*) were observed in these synapses. The action of insulin in this case may rather reflect its modulating effect on postsynaptic glutamate receptors and/or an increase in number of vesicles in the immediately releasable or primed pool at their presynapses. An increase of the mEPSCs frequency can also testify in favor of the latter, which is consistent with the previous reports, showing the stimulation of basal neurotransmitter release by insulin (Lee et al., 2011).

It has been suggested that insulin may target both pre- and postsynaptic membranes to affect basal synaptic transmission. For instance, it enhances the activity of postsynaptic NMDARs (N-methyl-D-aspartate receptors) in the synaptic membrane and mediates postsynaptic effects of the hormone (Skeberdis et al., 2001; van der Heide et al., 2005; Zhao et al., 2019). On the other hand, an increase in quantum content may reflect the stimulating effect of insulin on an increase in the number of releasable vesicles in these synapses, since *m* depends on *p* and *n* (the number of release sites). Apparently these changes do not affect the release probability (CV of eEPSCs and *p* did not change under insulin action).

Physiological and pathological conditions can influence insulin signaling and efficacy. For instance, patients with obesity and/or type 2 diabetes mellitus had lower insulin concentrations in the cerebrospinal fluid despite higher levels of this hormone in peripheral plasma (Heni et al., 2014). Obesity and inflammation can impair the transport of this hormone to the brain (Ketterer et al., 2011). As a result, changes in insulin signaling in the hippocampus can affect molecular mechanisms underlying synaptic plasticity and increase the risk of neurodegeneration and dementia (Kodl and Seaquist, 2008). Our previous results have shown that hypoinsulinemia causes significant weakening of synaptic activity in neural networks of cultured hippocampal neurons and decreases neurotransmitter release in their synapses (Shypshyna et al., 2021).

In order to address how insulin affects paired-pulse plasticity in the model of hypoinsulinemia, we specifically probed the effects of 100 nM insulin, as this concentration produced a robust response in the previous studies (Ghasemi et al., 2013; Zhao et al., 2019). Such insulin level is within concentration range that might occur locally in brain tissues *in vivo*. It was reported that normal physiological levels of insulin in circulating blood of adult rats is 180–240 pM (Havrankova et al., 1978, 1979). However, local accumulations of insulin can come from plasma circulation as well as activity-dependent insulin release from neurons and various glial cells (Ghasemi et al., 2013). Thus, *in vivo* insulin levels in brain tissues could be 10–100 times higher than in peripheral blood (Schechter et al., 1992; Duarte et al., 2012).

Our results have shown that against the background of hypoinsulinemia, insulin has a dual action on plasticity in hippocampal synapses. On the one hand, insulin did not have a significant effect on PPF of eEPSCs and did not change the binomial parameters *p* and *m* in these synapses; on the other hand, insulin changed PPD in the direction of increasing PPR and release probability *p* at these synapses.

It has been shown that PPF of eEPSCs is the most common form of paired-pulse plasticity at 50 ms interpulse interval (Debanne et al., 1996), which is the apparent reason for observing PPF under normoinsulinemia in our experiments. In addition, PPF of eEPSCs is most often observed at individual glutamatergic synapses with a low baseline release probability, whereas PPD occurred in synapses with a high release probability (Jiang et al., 2000). Therefore, we may predict the multidirectional effect of insulin on the short-term plasticity in hippocampal synapses under hypoinsulinemia depending on the baseline probability of glutamate release in them. Thus, in hypoinsulinemia, we observed both PPF and PPD, which may indicate the heterogeneity of hippocampal synapses in their resistance to insulin deprivation. Interestingly, in normoinsulinemia, insulin increased PPF, while in hypoinsulinemia such effect was not found in the PPF subgroup, whereas in the PPD subgroup, insulin appeared to decreased the PPD value. Thus, in hypoinsulinemia, the absence of insulin action on PPF neurons may indicate the development of insulin resistance, while the effect of insulin on PPD neurons indicates its ability to recover the form of plasticity to the control level in normoinsulinemia.

Thus, insulin has a modulating effect on short-term synaptic plasticity in hippocampal neurons, stimulating the glutamate release due to an increase in the quantal content in synapses of neurons under normoinsulinemia conditions. In our study under hypoinsulinemia at synapses with PPD, insulin recovered some properties of the paired-pulse plasticity to the normoinsulinemic level, including by increasing the probability of glutamate release in their synapses, but insulin did not have a significant effect on the parameters of paired-pulse plasticity at PPF synapses due to their probable insulin resistance.

## Data availability statement

The raw data supporting the conclusions of this article will be made available by the authors, without undue reservation.

## Ethics statement

The animal study was reviewed and approved by Animal Ethics Committee of the Bogomoletz Institute of Physiology (Kyiv, Ukraine).

## Author contributions

MS carried out the experiments, conceived and designed the analyses, collected the data, performed the analysis, and wrote the article. OK carried out the experiments, collected the data, and performed the analysis. SF and NV contributed to the study design and article editing. All authors contributed to the article and approved the submitted version.
